# Critically Exploring Vulnerability in Frail Older Adults Following a Fall

**DOI:** 10.1111/nup.70124

**Published:** 2026-09-27

**Authors:** Maria Kitsiou

**Affiliations:** ^1^ Department of Nursing School of Health Sciences University of Ioannina Ioannina Greece

**Keywords:** dignity, falls, frailty, nursing ethics, older adults, vulnerability

## Abstract

Vulnerability is widely invoked in healthcare, bioethics, and nursing, yet it remains poorly defined because universal human susceptibility, situational exposure to harm, and clinical frailty are frequently treated as interchangeable. This conceptual ambiguity is particularly significant following a fall, when older adults may be understood predominantly through physical risk, functional decline, and the need for protection. Through an interpretative analysis drawing on nursing philosophy, feminist bioethics, phenomenological accounts of embodiment, and nursing ethics, this paper critically examines the distinction between frailty and vulnerability in older adults following a fall. It argues that frailty describes a multidimensional clinical condition involving reduced physiological reserve, whereas vulnerability also emerges through lived experience, relationships, material resources, and institutional practices. A fall may therefore intensify vulnerability not only through bodily disruption but also through changes in dependence, decision‐making authority, and participation in everyday life. Nursing care occupies an ethically ambivalent position within this process: protective interventions may reduce avoidable harm while simultaneously restricting agency or generating pathogenic vulnerability. To address this tension, the paper develops the concept of responsive protection as an ethical orientation for post‐fall nursing care. Responsive protection requires protective practices to be negotiated with older adults, proportionate to the risks and burdens involved, and revisable as circumstances and capacities change. The paper concludes that safety, relational autonomy, and dignity should be understood as interdependent dimensions of an ethical nursing response to post‐fall vulnerability.

## 1 Introduction

Vulnerability is widely invoked in healthcare, bioethics, and nursing, yet its meaning remains conceptually contested. It may refer to a universal condition arising from human embodiment and dependence, or to an increased susceptibility to harm generated by particular personal, social, political, or institutional circumstances (Rogers et al. 2012; Sanchini et al. 2022). These perspectives are not necessarily opposing. Rather, they illuminate different sources and expressions of vulnerability. A lack of conceptual clarity, however, can result in older adults being described as inherently vulnerable solely because of their age or health status, obscuring the contextual and relational factors that shape their exposure to harm.

In later life, vulnerability is frequently conflated with frailty, although the concepts are not interchangeable. Frailty is a multidimensional and dynamic condition associated with declines in physiological reserve and function that reduce an individual's capacity to respond to everyday or acute stressors (Doody et al. 2023). Although its prevalence increases with age, frailty is not an inevitable consequence of ageing, nor is it exclusive to older adults. Vulnerability is a broader ethical concept that encompasses not only bodily susceptibility but also relational, social, and institutional exposure to harm (Rogers et al. 2012; Sanchini et al. 2022). Conflating the two concepts risks reducing older adults to their physiological deficits while overlooking how personal circumstances, relationships, and systems of care may intensify or alleviate vulnerability.

For the purposes of this paper, the term “older adults” refers to individuals aged 65 years and over, while recognising that chronological age alone does not adequately reflect the heterogeneity of ageing, health status, or functional ability (Sabharwal et al. 2015). The term ‘older adults’ is used instead of ‘the elderly’, in accordance with contemporary efforts to avoid language that may homogenise or stigmatise older adults on the basis of age (Murphy et al. 2022).

A fall is commonly approached as a clinical event requiring injury assessment, risk management, and rehabilitation. However, qualitative evidence indicates that its effects can extend beyond physical injury and pain to include restricted daily and social activities, reduced independence, fear, and helplessness (Jiang et al. 2024). A fall may therefore disrupt older adults' confidence in their bodies, their everyday routines, and their capacity to act independently. Havi Carel, a philosopher of medicine whose work examines illness and embodied vulnerability, draws on Merleau‐Ponty's concept of the body‐subject to describe human existence as embodied and situated and to explain how illness may affect autonomy and agency by disrupting what a person is able to do (Carel 2009). Applied to a fall, this phenomenological account suggests that the body may cease to be experienced as an unproblematic basis for action and become an explicit source of uncertainty and clinical concern. A fall can therefore be interpreted not only as physical harm but also as an embodied disruption that may intensify existing situational and relational vulnerabilities.

Although frailty is well established as a clinical concept (Doody et al. 2023) and vulnerability has been extensively examined within bioethics and moral philosophy (Rogers et al. 2012; Sanchini et al. 2022), the relationship between the two concepts following a fall remains insufficiently articulated within nursing philosophy. Conceptualising vulnerability predominantly in terms of clinical risk may reduce it to physical susceptibility and obscure the relational, social, and institutional conditions that shape exposure to harm (Rogers et al. 2012; Sanchini et al. 2022). This paper brings nursing philosophy into dialogue with bioethical and feminist accounts of vulnerability to argue that nursing care can either alleviate or intensify post‐fall vulnerability when risk management limits older adults' participation, agency, and autonomy. By clarifying the distinction between frailty and vulnerability, the paper develops an ethical account of post‐fall nursing care that extends beyond physical protection to include relational autonomy and dignity.

## 2 Purpose

This paper critically examines the distinction between frailty and vulnerability in older adults following a fall. It argues that a fall should not be understood solely as a manifestation or consequence of physical frailty, but as an event that may intensify situational and relational vulnerability. It further considers how nursing care can either mitigate these vulnerabilities by supporting agency, relational autonomy, and dignity or compound them through paternalistic and standardised responses. In doing so, the paper seeks to contribute to nursing philosophy by articulating an ethical understanding of post‐fall care that extends beyond physical risk management.

## 3 Theoretical Approach to Vulnerability

The distinction between different forms of vulnerability in aged care has been systematically examined by Sanchini et al. (2022). Their study is a systematic review of argument‐based ethics literature rather than a review of nursing research. Drawing on 38 publications published between 1984 and 2020 and identified through searches designed to capture biomedical, philosophical, bioethical, and anthropological literature, the review examined the meaning, foundations, and ethical uses of vulnerability in aged care. Sanchini et al. identified two broad dimensions: basic human vulnerability, which arises from embodiment, finitude, and dependence, and situational vulnerability, which is shaped by contingent personal, social, political, and institutional conditions. In this paper, their review is used as a bioethical mapping of vulnerability in aged care rather than as a nursing theory. Its categories provide a starting point that is subsequently brought into dialogue with nursing philosophy and feminist accounts of autonomy, dependence, and care.

## 4 Basic and Ontological Vulnerability

Basic human vulnerability refers to the susceptibility arising from embodiment, finitude, dependence, and the possibility of illness or harm that is shared by all human beings (Rogers et al. 2012; Sanchini et al. 2022). Within feminist bioethics, Rogers et al. (2012) distinguish this universal dimension from vulnerabilities produced by specific contexts and relationships, emphasising that conceptual clarity is necessary to determine both the sources of vulnerability and the responsibilities it generates. They also distinguish between dispositional and occurrent vulnerability: a person may be susceptible to harm without currently experiencing it, whereas occurrent vulnerability involves an existing state of vulnerability. Applied to post‐fall care, this distinction suggests that a fall may transform a previously dispositional susceptibility into an occurrent vulnerability through injury, functional limitation, or increased dependence. This transition is neither inevitable nor uniform, because its meaning and effects remain shaped by older adults' circumstances and available support. They further argue that vulnerability should not be positioned in opposition to autonomy. Drawing on relational approaches, they describe autonomy as a socially constituted capacity that develops and is exercised through interpersonal relationships and social conditions and may therefore be supported or undermined by practices and institutions.

Within nursing philosophy, Derek Sellman conceptualises nursing as a response to ‘more‐than‐ordinary vulnerability’. His argument begins from the premise that all human beings are vulnerable but that receiving nursing care can expose individuals to additional risks and dependencies that they may be unable to mitigate without the actions of others (Sellman 2005). Havi Carel, a philosopher of medicine, extends this discussion by drawing attention to the embodied and subjective experience of vulnerability in illness and to the existential exposure involved in encountering the suffering of others (Carel 2009). Read together, these perspectives shift attention from whether older adults are vulnerable to how vulnerability is experienced, intensified, and responded to following a fall. Sellman foregrounds the need for assistance when risks cannot be mitigated independently, while Carel draws attention to the embodied experience through which disruption and dependence may be lived. Rogers et al. (2012) further complicate the assumption that increased dependence necessarily diminishes autonomy, arguing that autonomy is socially constituted and may be supported or undermined by the relationships and conditions through which care is provided. In the post‐fall context, vulnerability therefore concerns not only exposure to harm but also how bodily disruption, dependence, and care relationships shape older adults' possibilities for agency.

## 5 Situational Vulnerability

Situational vulnerability arises from contingent personal, social, political, and institutional circumstances rather than from embodiment alone (Rogers et al. 2012; Sanchini et al. 2022). In aged care, these circumstances may include illness, functional decline, social isolation, limited access to healthcare, economic disadvantage, ageism, and dependence on formal or informal support. Such factors do not make older adults uniformly vulnerable. Instead, they interact with older adults' individual capacities, relationships, resources, and environments, producing different forms and degrees of exposure to harm. Situational vulnerability is therefore dynamic: it may emerge, intensify, diminish, or change as personal circumstances and available forms of support change over time.

A particularly important form of situational vulnerability is pathogenic vulnerability, which may arise when social, institutional, or interpersonal responses exacerbate existing vulnerabilities or generate new ones. This can occur when measures intended to provide protection instead increase powerlessness, loss of control, or loss of agency (Rogers et al. 2012). Within nursing care, this possibility requires attention to how risk‐management practices are implemented. Interventions may reduce immediate physical risk while simultaneously restricting older adults' participation or reinforcing assumptions of incapacity. The ethical significance of situational vulnerability therefore lies not only in identifying exposure to harm but also in examining whether care relationships and institutional practices alleviate existing vulnerability or inadvertently intensify it.

## 6 Sellman's Typology of Risk

Sellman (2005) approaches vulnerability through a tripartite typology of risk based on whether, and by whom, a particular risk can be reduced. His categories do not represent progressively increasing ‘levels’ of vulnerability. Rather, they distinguish risks according to the possibilities and limits of individual, relational, and institutional action.

1. The first category includes risks that can be reduced through the actions of individuals themselves. In the post‐fall context, these may include engaging in rehabilitation, using appropriate mobility aids, or adopting recommended safety practices. However, older adults' capacity to take such actions may be constrained by frailty, pain, cognitive impairment, limited resources, or inaccessible environments.
2. The second category includes risks that can be reduced through the actions of others, including family members, nurses, other healthcare professionals, and institutional regulations. Following a fall, these actions may include clinical assessment, medication review, rehabilitation support, environmental modification, care coordination, and organisational measures designed to promote safety. This category demonstrates that the reduction of vulnerability cannot always be achieved through individual responsibility and may depend on the availability and quality of relational and institutional support.
3. The third category includes risks that remain despite the actions of individuals or others, including institutional measures and regulations. These risks reflect the limits of prevention and protection: even when older adults, healthcare professionals, and institutions act appropriately, uncertainty and the possibility of harm cannot be eliminated entirely.

Applied to post‐fall care, Sellman's typology clarifies that vulnerability is shaped not only by the presence of risk but also by older adults' capacity to respond to it and by the actions of those on whom they depend. Nursing care has an important protective role in relation to the second category of risk, but Sellman's third category also establishes an ethical limit to the expectation that all vulnerability can be prevented or controlled. Nursing should therefore aim to reduce avoidable harm without treating vulnerability as a condition that can, or should, be eliminated entirely.

## 7 Distinguishing Vulnerability From Frailty

Frailty and vulnerability are related but conceptually distinct. Frailty is a multidimensional and dynamic clinical condition characterised by declining reserve and function across multiple physiological systems, resulting in a reduced capacity to respond to everyday or acute stressors (Doody et al. 2023). It is associated with adverse outcomes, including falls, hospitalisation, disability, and mortality. Although frailty is more prevalent in later life, it is neither an inevitable consequence of ageing nor exclusive to older adults (Doody et al. 2023).

Vulnerability has a broader ethical and relational meaning. It includes the universal susceptibility associated with embodiment and dependence, as well as situational forms of exposure shaped by relationships, social conditions, resources, and institutional practices (Rogers et al. 2012; Sanchini et al. 2022). Frailty may increase vulnerability by reducing physiological resilience and older adults' capacity to respond independently to risk. However, vulnerability cannot be inferred from frailty alone. Older adults with similar levels of frailty may experience substantially different forms and degrees of vulnerability depending on their social support, material resources, care relationships, living environments, and opportunities to participate in decisions.

Maintaining this distinction has important implications for nursing care. When frailty and vulnerability are treated as interchangeable, older adults may be understood primarily through deficits and risk, potentially encouraging overprotection and assumptions of incapacity. A relational understanding of autonomy offers an alternative approach by recognising that dependence does not necessarily negate autonomy and that agency can be supported through appropriate relationships and social conditions (Rogers et al. 2012). Nursing care should therefore respond to frailty through appropriate clinical assessment and risk reduction while also examining the relational and institutional conditions that may alleviate or intensify vulnerability following a fall.

## 8 Dimensions of Vulnerability in Older Adults After a Fall

A fall does not create vulnerability in isolation, nor does it affect all older adults in the same way. Rather, it can expose, intensify, and reorganise vulnerabilities that arise through the interaction of embodiment, personal circumstances, relationships, and institutional conditions. The distinction between basic human and situational vulnerability is therefore analytically useful, but it should not be understood as a division between two separate or fixed states (Rogers et al. 2012; Sanchini et al. 2022). Basic vulnerability makes bodily injury and dependence possible, while situational conditions influence how the fall is experienced, what resources are available, and whether older adults are able to retain meaningful control over their lives.

The physical consequences of a fall acquire ethical significance when they affect older adults' agency and relationship with their bodies. Injury, pain, and functional decline may restrict activity, but the experience of vulnerability cannot be inferred from physical impairment alone. Qualitative evidence shows that falls may also be followed by fear, helplessness, reduced independence, and restrictions in daily and social activities (Jiang et al. 2024). These experiences suggest that a fall can unsettle bodily confidence and alter how older adults understand their capacities and future possibilities. The fall therefore becomes more than a clinical event: it may mark a transition in which the body is experienced as less predictable and everyday action becomes increasingly shaped by anticipation of further harm.

Dependence following a fall is similarly ethically ambiguous. Reliance on family members, caregivers, or healthcare professionals does not necessarily diminish autonomy. When care relationships provide appropriate support, information, and opportunities for participation, they may create the social conditions through which older adults can exercise autonomy despite physical limitations (Rogers et al. 2012). However, when dependence is interpreted as incapacity, protective practices may silence older adults' preferences and transfer control to others. The same intervention can therefore be experienced as supportive or restrictive depending on how decisions are made and whether older adults' agency is recognised.

Institutional responses are central to this process. Risk assessment, mobility restrictions, rehabilitation planning, and discharge decisions may reduce preventable harm, but they also define which risks are considered acceptable and whose judgement is treated as authoritative. When safety is pursued without meaningful participation, care may produce pathogenic vulnerability by increasing powerlessness or dependence through measures intended to provide protection (Rogers et al. 2012). Post‐fall vulnerability should therefore be understood not as an attribute located within older adults, but as a relational and evolving process shaped by the interaction between bodily disruption, lived experience, dependence, and the organisation of care.

## 9 Nursing Care as a Response to Vulnerability

Post‐fall nursing care is shaped by a fundamental ethical tension: older adults may require protection from further harm while also needing to retain control over decisions affecting their bodies and everyday lives. Sellman's (2005) understanding of nursing as a response to ‘more‐than‐ordinary vulnerability’ establishes a legitimate protective responsibility, particularly when older adults cannot reduce particular risks without professional or institutional support. Protection, however, is not an uncomplicated ethical good. When vulnerability is interpreted primarily as incapacity, the authority to define acceptable risk may shift from older adults to healthcare professionals. The protective function of nursing may then reduce immediate physical danger while simultaneously narrowing the space in which older adults can exercise agency.

This tension demonstrates why vulnerability cannot be understood as a property located exclusively within older adults. Research examining vulnerability from the perspectives of both older adults and nurses offers two intersecting but distinct accounts. Older adults have associated vulnerability with the possibility of harm, social positioning, and threats to dignity, while also recognising protection, strength, and the capacity for development (Sarvimäki and Stenbock‐Hult 2016). Nurses caring for older adults have similarly described vulnerability as both a burden and a resource, involving emotional exposure, moral indignation, courage, and processes of maturing and personal development (Stenbock‐Hult and Sarvimäki 2011). Read together, these findings suggest that the nursing encounter is characterised by mutual vulnerability, but not by equal power. Nurses may be emotionally and morally exposed through care, yet they also possess professional and institutional authority that can shape older adults' mobility, treatment, discharge, and participation in decisions.

The ethical significance of nursing care therefore lies not simply in recognising vulnerability but in determining how that recognition is translated into practice. Recognition may generate attentiveness and responsive support, but it may also legitimise surveillance, restriction, or substituted decision‐making when vulnerability is equated with inability. This ambiguity becomes particularly visible after a fall. Fear, reduced confidence, functional limitation, and dependence may increase older adults' need for assistance (Jiang et al. 2024), but they do not establish that older adults are unable to interpret their circumstances or participate meaningfully in decisions. Dependence can support autonomy when relationships and resources provide the social conditions through which agency can be exercised; it can undermine autonomy when assistance becomes control (Rogers et al. 2012).

Gastmans' (2013) framework of dignity‐enhancing nursing care helps clarify the normative direction of this response. By connecting lived experience, interpretative dialogue, and ethical standards, Gastmans positions dignity not as an abstract value added to technically competent care but as a criterion by which care itself should be evaluated. In the post‐fall context, interpretative dialogue requires nurses to understand not only what risks are clinically present but also what mobility, independence, assistance, and acceptable risk mean to older adults. Yet dialogue alone is insufficient if institutional routines have already determined the available choices. Dignity‐enhancing care must therefore address both the interpersonal quality of the nursing relationship and the organisational conditions that structure older adults' participation.

Nursing care can consequently alleviate and produce vulnerability at the same time. A mobility restriction may prevent an immediate fall while increasing dependence; assistance may enable activity or replace it; risk assessment may facilitate appropriate support or transform older adults into categories of risk. The ethical task is not to choose between safety and autonomy as if they were mutually exclusive, but to examine how protection can be provided without unnecessarily displacing agency. Post‐fall nursing care responds adequately to vulnerability when clinical risk reduction, interpretative understanding, relational autonomy, and dignity are treated as interdependent rather than competing aims.

## 10 Ethical Considerations: Towards Responsive Protection

The ethical significance of a fall lies not only in the harm it causes or the possibility that it may recur. A fall can alter the distribution of knowledge, authority, and decision‐making within care. Older adults who previously determined their own movements and routines may become subject to professional assessments of capacity, safety, and acceptable risk. Clinical knowledge is necessary for identifying injury and anticipating harm, but it can also acquire interpretative authority over what the fall means and how older adults should subsequently live. The central ethical problem is therefore not simply how nursing should respond to vulnerability, but how it can do so without transforming vulnerability into a justification for displacing older adults' agency.

Sellman's (2005) account makes protection a legitimate and fundamental function of nursing because some risks cannot be reduced without the actions of others. Yet the moral obligation to protect does not determine, by itself, what protection should look like or how far it may extend. Protection can respond to vulnerability, but it can also produce a moral asymmetry in which professionals define older adults’ needs while older adults become the objects of protective action. The ethical difficulty is that the same intervention may be both beneficial and restrictive: assistance can enable movement or replace it, supervision can create confidence or surveillance, and mobility restrictions can prevent immediate harm while contributing to dependence and functional decline. Protection must therefore be evaluated not only according to the harm it prevents but also according to the relationships and forms of agency it creates.

A relational understanding of autonomy offers a way of interpreting this ambiguity without opposing autonomy to dependence. Rogers et al. (2012) argue that autonomy is a socially constituted capacity that develops and is exercised through interpersonal relationships and social conditions. Dependence following a fall may consequently support autonomy when assistance expands older adults' opportunities to act, communicate preferences, and pursue valued activities. The same dependence becomes autonomy‐limiting when support is conditional on compliance or when others assume authority over decisions that older adults remain capable of making. This distinction is reinforced by evidence that older adults understand vulnerability not only through harm and threats to dignity but also through protection, strength, and the capacity for development (Sarvimäki and Stenbock‐Hult 2016). Vulnerability should therefore not be interpreted as evidence that agency has disappeared; it may instead identify the conditions under which agency requires support.

This paper proposes responsive protection as an ethical orientation for post‐fall nursing care. Responsive protection differs from protection imposed solely on the basis of professionally defined risk. It begins with the recognition that both clinical assessment and older adults' lived knowledge are necessary for understanding what is at stake. It is negotiated, because the aims and acceptable burdens of intervention should be interpreted with older adults rather than determined exclusively for them. It is proportionate, because the restriction of mobility or choice should not exceed what is reasonably required to address a specific risk. It is also revisable, because vulnerability, capacity, recovery, and acceptable risk are not static. Responsive protection does not abandon nursing's responsibility to prevent avoidable harm; it requires that protection remain accountable to older adults' agency. Its distinctive contribution lies in integrating Sellman's (2005) account of protective responsibility, the relational account of autonomy employed by Rogers et al. (2012), and Gastmans' (2013) account of dignity‐enhancing care within a single ethical framework tailored to post‐fall nursing care. In doing so, responsive protection reframes safety and agency not as competing obligations but as interdependent criteria by which protective practices should be designed and evaluated.

For example, when an older adult wishes to mobilise independently despite an increased risk of another fall, conventional risk minimisation may favour blanket restriction or continuous supervision. Responsive protection instead requires the nurse to explore what mobility means to the older adult, negotiate proportionate forms of assistance, and revise the plan as recovery, confidence, and circumstances change. The question is therefore not whether safety or autonomy should prevail, but how protection can reduce avoidable harm while sustaining agency.

This orientation also shifts ethical attention from individual encounters to institutional conditions. Fall‐prevention protocols, documentation requirements, staffing arrangements, and organisational concerns about adverse events influence which options nurses can offer and how much risk institutions are prepared to tolerate. These structures are not ethically neutral. When institutional risk avoidance limits meaningful participation or converts temporary support into continuing restriction, care may generate pathogenic vulnerability by exacerbating existing vulnerabilities or creating new ones through the very mechanisms intended to provide protection (Rogers et al. 2012). Nurses may recognise older adults' preferences while remaining constrained by systems that privilege measurable safety outcomes over mobility, recovery, or participation. Ethical responsibility must therefore include critical examination of the organisational conditions through which protection is delivered.

Dignity provides the normative standard by which responsive protection should ultimately be evaluated. Gallagher (2004) shows that dignity concerns both inherent human worth and the practical ways in which that worth is recognised or violated. Gastmans (2013) similarly understands nursing care as a response to vulnerability directed towards maintaining, protecting, and promoting dignity. In post‐fall care, dignity is not secured through respectful language alone if older adults' interpretations and choices have no influence on what occurs. It is enacted when older adults remain recognised as participants in care rather than being reduced to frailty, injury, or fall risk. Nursing responds ethically to post‐fall vulnerability when protection reduces avoidable harm while preserving the relational and institutional conditions through which older adults can continue to exercise agency.

## 11 Final Considerations

Distinguishing frailty from vulnerability does more than resolve a conceptual ambiguity; it changes what nursing is able to perceive following a fall. When the two concepts are treated as interchangeable, reduced physiological reserve, functional decline, and increased risk can become the dominant explanation of older adults' circumstances. Although assessment, prevention, and protection remain necessary, they provide only a partial account of what a fall may produce. Vulnerability directs attention beyond physiological susceptibility towards the relationships, resources, interpretations, and institutional conditions through which older adults experience and respond to bodily change.

A fall should therefore be understood neither solely as a physical event nor as a universal symbol of human fragility. Its ethical significance emerges through the interaction between bodily disruption and the circumstances in which that disruption is interpreted and managed. The same injury may have different implications depending on access to supportive relationships, rehabilitation, material resources, and meaningful participation in decisions. Dependence likewise has no fixed ethical meaning: it may enable recovery and agency when support expands possibilities for action, or deepen vulnerability when assistance becomes substitution and protection becomes control. Post‐fall vulnerability is consequently not a stable characteristic located within older adults, but a relational process that can be intensified or alleviated through care.

This understanding places nursing in an ethically complex position. Nursing does not simply encounter vulnerability as something that already exists; through assessment, language, routines, restrictions, and relationships, it participates in shaping how vulnerability is experienced and which possibilities remain available to older adults. Its protective function is therefore both necessary and morally ambivalent. Protection may prevent avoidable harm, but it may also transfer authority away from older adults or reinforce an identity organised around frailty and risk. Ethical care must consequently be evaluated not only by the safety it achieves but also by the forms of dependence, participation, and agency it produces.

Responsive protection offers a way of addressing this moral ambiguity by integrating protective responsibility, relational autonomy, and dignity within a framework tailored to post‐fall nursing care. It requires protection to be negotiated through interpretative dialogue, proportionate to the risks and burdens involved, and revisable as older adults’ circumstances and capacities change. It does not seek to eliminate vulnerability or establish a universal balance between safety and autonomy. Rather, it requires protective practices to reduce avoidable harm while remaining accountable to older adults’ agency.

Responsive protection also makes visible the institutional dimension of nursing ethics. The possibilities available within caring relationships are shaped by staffing, organisational routines, professional hierarchies, documentation practices, and institutional approaches to risk. Supporting agency therefore cannot remain the responsibility of individual nurses alone. Healthcare institutions must examine whether structures designed to promote safety allow sufficient space for dialogue, individual judgement, and shared decision‐making. Otherwise, well‐intentioned systems of protection may reproduce the pathogenic vulnerabilities they seek to prevent.

The contribution of nursing philosophy is not to replace clinical knowledge of falls or frailty, but to examine the assumptions through which that knowledge is translated into care. It asks whose interpretation of risk becomes authoritative, which forms of dependence are enabled, and whether older adults remain recognised as agents within the practices intended to protect them. Post‐fall nursing care becomes ethically adequate when physical safety, relational autonomy, and dignity are understood as interdependent dimensions of a response to vulnerability. The moral task of nursing is therefore not only to protect older adults from another fall, but also to ensure that protection does not itself become another source of vulnerability.

## Funding

The author has nothing to report.

## Ethics Statement

The author has nothing to report.

## Conflicts of Interest

The author declares no conflicts of interest.

## Data Availability

The author has nothing to report.
